# Enhancing nicotine replacement therapy usage and adherence through a mobile intervention: Secondary data analysis of a single-arm feasibility study in Mexico

**DOI:** 10.18332/tid/120076

**Published:** 2020-05-04

**Authors:** Francisco Cartujano-Barrera, Rosibel Rodríguez-Bolaños, Evelyn Arana-Chicas, Katia Gallegos-Carrillo, Yvonne N. Flores, Gloria Pérez-Rubio, Ramcés Falfán-Valencia, Edward F. Ellerbeck, Luz Myriam Reynales-Shigematsu, Ana Paula Cupertino

**Affiliations:** 1 Department of Cancer Prevention and Control, Hackensack University Medical Center, Hackensack, United States; 2 Departamento de Investigación sobre Tabaco, Instituto Nacional de Salud Pública, Cuernavaca, Mexico; 3 Unidad de Investigación Epidemiológica y en Servicios de Salud, Delegación Morelos, Instituto Mexicano del Seguro Social, Cuernavaca, Mexico; 4 Department of Health Policy and Management, UCLA Fielding School of Public Health, Los Angeles, United States; 5 Laboratorio HLA, Instituto Nacional de Enfermedades Respiratorias, Ciudad de Mexico, Mexico; 6 Department of Preventive Medicine and Public Health, University of Kansas Medical Center, Kansas City, United States; 7 James P. Wilmot Cancer Institute, University of Rochester Medical Center, Rochester, United States

**Keywords:** behavioral counseling, cessation, global health, pharmacotherapy, public policy

## Abstract

**INsTRODUCTION:**

Nicotine Replacement Therapy (NRT) is an effective treatment for smoking cessation. However, medication usage and adherence remain a challenge that contributes to low smoking cessation rates. In Mexico, 8 in 10 smokers are interested in quitting. However, only 6% of Mexican smokers use medication for smoking cessation. The objective of this study is to assess the feasibility and acceptability of a mobile health (mHealth) intervention to increase usage and adherence of NRT in Mexico.

**METHODS:**

The study involves a secondary data analysis. Forty smokers were recruited to participate in a single-arm pilot study. Participants received an mHealth intervention that uses tablet-based decision support software to drive a 12-week text messaging smoking cessation program and pharmacotherapy support. The intervention allows two-way interactivity text messaging between participants and a tobacco treatment specialist. NRT was offered to participants in accordance with practice guidelines in Mexico. Outcome measures included utilization of NRT, text messaging interactivity with the program, and biochemically verified abstinence at 12 weeks.

**RESULTS:**

Thirty smokers met the criteria for use of NRT. Average age of participants was 38.1 years (SD=10.7), and they were primarily male (56.7%) with at least an undergraduate degree (60%). All participants requested NRT at baseline, and 60% requested a refill at week 4. During the 12-week intervention period, participants sent 620 messages to the program (mean=20.6, SD=18.34) of which 79 messages (12.7%) were related to NRT. Three themes were identified in the messages related to NRT: enthusiasm, instructions, and side effects. At 12 weeks, 40% of participants reported using NRT <75% of the days. Finally, 30% of participants (9/30) were biochemically verified abstinent using intention-to-treat analysis at 12 weeks.

**CONCLUSIONS:**

An mHealth intervention appears to offer a promising strategy to increase usage and adherence of NRT in Mexico. Additional testing as a formal randomized clinical trial appears warranted.

## INTRODUCTION

Nicotine Replacement Therapy (NRT) is an effective and safe treatment for smoking cessation^1^. NRT reduces the physiological and psychomotor withdrawal symptoms often experienced while attempting to quit smoking and increases the rate of long-term quitting by 50% to 60%^1^. Since 1996, the Food and Drug Administration (FDA) has approved over-the-counter (OTC) availability of NRT medications, such as nicotine patches and gum^2^. OTC availability of NRT has shown to increase access to and utilization of treatment^3^.

However, NRT usage and adherence remain a challenge that contributes to low smoking cessation rates. The 2015 Global Adult Tobacco Survey found that utilization of cessation aids is between 2% (Egypt and Ukraine) to 26% (Indonesia) among smokers who attempted to quit^4^. Moreover, studies have shown that individuals receiving pharmacotherapy for tobacco dependence often use it at a lower dose and for less time than optimal, as evidence suggests^5-7^. For instance, approximately half of smokers using NRT adhere to the recommended duration of treatment^5-7^.

Compliance with the recommended treatment duration is significant because evidence indicates that greater medication adherence is associated with greater abstinence^5-7^. A number of factors have been shown to limit adherence to NRT. A recent review of 48 studies by Pacek et al.^8^ examined reasons for suboptimal usage of NRT and other cessation pharmacotherapy among smokers and ex-smokers. This review distinguished non-preventable factors (e.g. comorbidities, tobacco dependence, and sociodemographic characteristics) from preventable factors (e.g. beliefs, attitudes, and psychosocial characteristics) for suboptimal usage of pharmacotherapy^8^. Overcoming the burden of tobacco use, including low utilization and non-adherence of pharmacotherapy for smoking cessation, demands affordable, accessible, and effective solutions – such as mobile health (mHealth) interventions defined as the provision of health services and information via mobile technologies such as mobile phones, tablet computers and Personal Digital Assistants (PDAs), and wearable devices such as smart watches^9^.

A systematic review examined the effectiveness of interventions to improve usage and adherence to medications for nicotine dependence and found that adherence interventions led to marginal improvements^10^. A paucity of studies, included in the review, utilized mHealth technologies: two studies included phone calls and one study consisted of a smartphone app; none of the studies utilized text messages^10^.

The present pilot study assessed the feasibility and acceptability of a smoking cessation mHealth intervention to increase usage and adherence of NRT in Mexico. This intervention allows two-way interactive text messaging between participants and a tobacco treatment specialist. In Mexico, an upper middle-income country^11^, 8 in 10 smokers are interested in quitting^4,12^. However, only 6% of Mexican smokers take advantage of pharmacotherapy for smoking cessation^4,12^. Factors that account for the low usage of pharmacotherapy in Mexico include healthcare providers who fail to initiate cessation treatment^13^ and government insurance for low-income Mexicans that does not cover pharmacotherapy^14,15^. Low availability of NRT is another challenge in Mexico^15^. An mHealth intervention that provides access to NRT at no cost and manages use and side effects might increase usage and adherence of NRT among Mexican smokers.

## METHODS

### Research design

The present study includes a secondary data analysis of a single-arm feasibility study in Mexico. The parent pilot study, described in detail elsewhere, assessed the feasibility and acceptability of an innovative, personalized and interactive smoking cessation mobile intervention developed for Mexican smokers^16^.

### Participants

In 2017, 40 smokers were recruited using a multimedia campaign (Facebook and radio) to participate in a smoking cessation single-arm pilot study. The parent pilot study describes the recruitment methods in more detail^16^. Inclusion criteria for participants were: 1) Mexican origin, 2) aged ≥18 years, 3) smoked for at least 6 months, 4) smoked at least 3 days per week, 5) interested in quitting within the next 30 days, 6) had a cell phone with text messaging capability, and 7) willing to complete surveys at baseline and at follow-up at 12 weeks. Exclusion criteria were: 1) planning to move within the next 6 months, 2) consuming other forms of tobacco, using an electronic nicotine delivery system or drugs that are illegal in Mexico (e.g. cannabis and cocaine), and 3) having another household member enrolled in the study. Written informed consent was obtained from each participant. The Human Subjects Committee of the Instituto Nacional de Salud Púa approved and monitored the study procedures.

### Intervention

*Vive sin Tabaco… Decídete!* (Live Without Tobacco?… Decide!) is a smoking cessation mHealth intervention that encompasses three integrated components: 1) a tablet-based software that collects personal smoking-related information to support the development of an individualized quit plan; 2) a 12-week individually-tailored text messaging program with interactive capabilities that includes educational information, behavioral strategies, and motivational messaging driven by information obtained from the tablet-based software; and 3) pharmacotherapy support, when applicable. Details of the intervention are described in detail elsewhere^16^.

Regarding pharmacotherapy management, the tablet-based software featured short video clips and narrated graphics on how NRT can support abstinence and prompted interested participants to request pharmacotherapy, if indicated. The text messaging program, based on participants’ selected quit dates, targeted modifiable preventable characteristics for suboptimal usage of NRT described by Pacek et al.^8^ (Table 1). The text messages also prompted pharmacotherapy adherence via weekly reminders. Moreover, participants received messages to prompt them to text the program for help to troubleshoot side-effects, manage nicotine withdrawal symptoms, and address other concerns related to cessation. A certified tobacco treatment specialist responded to participant-generated text messages within 48 hours.

Nicotine patches were offered to participants in accordance with clinical practice guidelines for treating smokers in Mexico: only daily smokers who smoked six or more cigarettes per day (CPD) were eligible to use nicotine patches^17,18^. Nicotine patches were contraindicated and not offered to participants who: 1) had a heart attack in the last 30 days, 2) had a stroke in the last six months, 3) had been diagnosed with arrhythmia or tachycardia, 4) had uncontrolled hypertension, or 5) were using warfarin. Participants who smoked ≥10 CPD and had no contraindications were offered ten weeks of nicotine patches: 21 mg nicotine patches to be used during the first six weeks, followed by 14 mg nicotine patches for two weeks, and 7 mg patches for the last two weeks. Participants who smoked 6–9 CPD and had no contraindications, were offered eight weeks of nicotine patches: 14 mg nicotine patches to be used during the first six weeks, followed by 7 mg patches for the last two weeks. At baseline, each eligible participant received a 4-week supply if they were interested in using NRT. Participants were prompted to start using their nicotine patches on their selected quit-date, which was set at their baseline visit. In the beginning of the second week of the intervention, participants received text message prompts to request more NRT. If a participant indicated interest, a 4- or 6-week supply was shipped to their home at no cost.

**Table 1:** Examples of text messages targeting preventable characteristics for suboptimal use of NRT

*Preventable characteristic*	*Day*	*Messages*
Smoker’s belief that NRT does not help with cessation	Day 1 Post-enrollment	Did you know that using nicotine patches doubles your chance to quit smoking? It is scientifically proven, get ready to use it!
	Day 3 Post-enrollment	Daily use of Nicotine Replacement Therapy (NRT) helps your body adjust to your new cigarette-free life.
Smoker’s fear of becoming dependent by using NRT	Day 2 Post-enrollment	How do nicotine patches work? Patches give your body nicotine through the skin in a safe way and without causing addiction.
	Day 8 Post-enrollment	‘At first, I was afraid to put more nicotine into my body by using the nicotine patch, but then I realized NRT helped me with my cigarette cravings and I could finally quit’ Josue, 33 years old.
Smoker’s lack of knowledge regarding how to correctly use NRT	Pre-quit Day	Put a nicotine patch on your arm tomorrow when you wake up. This will keep you calm during the day, use it!
	Day 54 Post-quit	You are about to complete the second stage of your process (14 mg patches). Two more days and you will be using the 7 mg nicotine patches. Are you ready?
Smoker’s lack of knowledge regarding nicotine withdrawals	Pre-quit Day	Tomorrow you will probably feel uncomfortable or have some anxiety after quitting. This is known as Abstinence Syndrome. This is normal since your body is eliminating toxins from cigarettes. Using nicotine patches will help decrease these discomforts.
Advice from a healthcare provider to continue using NRT	Day 4 Post-quit	Good morning, we are going to conquer a new day. Keep using the nicotine patches. Text us with whatever you need!
	Day 68 Post-quit	Did you put your nicotine patch on already? Three more patches and you will have finished your treatment. Congratulations [Name]!
Smoker’s desire to test their ability to remain quit without NRT	Day 22 Post-quit	Keep using the patch for 7 more weeks. Completing your treatment is necessary to quit cigarettes.
	Day 40 Post-quit	We don’t recommend you to go without the patches for one day. It’s not time for that yet. Is important for your body to slowly get less and less nicotine until you don’t need any.

### Measures

Research staff conducted an assessment, in person, at baseline and at follow-up at 12 weeks. The baseline survey collected sociodemographic information (e.g. age, gender, education level, marital status, and type of health insurance). Other smoking behaviors variables collected were: physical nicotine dependence (Fagerström Test for Nicotine Dependence^19^), CPD, and number of previous quit attempts. Participants’ interactions with the program via text messages were monitored throughout the entire 12-week intervention and retrospectively analyzed to explore the participants’ experiences of pharmacotherapy management. At the follow-up visit at 12 weeks, the following were assessed: cessation outcomes and pharmacotherapy use, delivery, and side effects. The cessation outcome was cotinine-verified 7-day point prevalence abstinence (not smoking any cigarettes in the past seven days). This was biochemically verified using urinary cotinine testing, with a cut-off of 200 ng/mL cotinine^20,21^. If the participant was still using NRT, smoking abstinence was verified using exhaled carbon monoxide with a cut-off at 6 ppm^20,21^. The cessation rate was calculated using an intent-to-treat analysis, in which participants who were lost to follow-up were considered smokers.

At the end of the intervention, participants self-reported daily patch-usage data. As done by Schlam et al.^22^, we calculated the percentage of days participants used the patch with the number of days of prescribed medication (8 or 10 weeks depending their baseline CPD) as the denominator. We also assessed the method of application for NRT. This included open-ended questions such as ‘How do you use the nicotine patches?’ with ‘I used one nicotine patch a day in a clean, dry and hairless area of my body’ as the correct answer.

### Analysis

For the purposes of this study, only smokers who were eligible to use NRT were included in the analysis. We calculated simple frequencies for categorical variables and means and standard deviations for continuous variables. Participants’ text messages were imported into Microsoft Excel to retrospectively identify messages related to NRT for qualitative thematic analysis. Two coders independently analyzed the data using a process of inductive thematic analysis^23^. Subsequently, themes were grouped into coding themes and a code map was developed^24^. The two coders met weekly and compared findings to identify similarities and differences with the codes. Discrepancies were addressed by a third coder.

## RESULTS

Thirty smokers were eligible to use NRT. Participants’ age at baseline (n=30) ranged from 20 to 59 years (mean=38.1; SD=10.7). Seventeen (56.7%) participants were men, 50% were single, 60% had college or postgraduate education, and 83.3% had health insurance coverage. Less than half of the participants (40%) were light smokers (smoked <10 CPD) and, according to the Fagerström test, 60% of participants reported low levels of nicotine dependence (Table 2).

**Table 2:** Baseline characteristics of participants (N=30)

*Characteristics*	*n (%)*
**Age**, mean ± SD	38.1 ± 10.7
**Sex**	
Male	17 (56.7)
Female	13 (43.3)
**Education level**	
Less than high school graduate	3 (10.0)
High school graduate	5 (16.7)
Technical school	4 (13.3)
College graduate	12 (40.0)
Postgraduate	6 (20.0)
**Marital status**	
Married/cohabitating	12 (40.0)
Single	15 (50.0)
Divorced/separated/widowed	3 (10.0)
**Health coverage**	
Instituto Mexicano del Seguro Social^a^	19 (63.3)
Instituto de Seguridad y Servicios Sociales de los Trabajadores del Estado^b^	6 (20.0)
None	5 (16.7)
**Daily cigarette smoking**	
6–9	12 (40.0)
10–19	12 (40.0)
≥20	6 (20.0)
**Fagerström Test for Nicotine Dependence**	
Low	18 (60.0)
Moderate	9 (30.0)
High	3 (10.0)
**Quit attempt in previous year**	
Yes	16 (53.3)
No	14 (46.7)

a Mexican Social Security Institute.

b Institute for Social Security and Services for State Workers.

All participants requested NRT at baseline. At week 4, 18 (60%) participants requested a refill of NRT. At 12 weeks, 25 participants completed the follow-up survey (83.3% follow-up rate). All participants requesting NRT by mail reported that they received it. Based on the distribution of the variable ‘percentage of days used the patches’, we created four usage categories: used the patch <75% of days (40% of the sample); 50–75% of days (36% of the sample); 25–50% of days (20% of the sample); and >25% of days (4% of the sample). When asked about the method of application of nicotine patches, all participants (100%) reported using it correctly. When asked about side effects, four participants (16%) reported experiencing dizziness, three (12.5%) reported experiencing nausea, two (8%) reported experiencing headaches, and one (4%) reported having nightmares. Using intention-to-treat analysis, biochemically verified abstinence at 12 weeks was 30% among the participants (9/30).

During the 12-week intervention period, participants sent an average of 20.6 (Range: 0–68; SD: 18.34) text messages. Out of the 620 messages that participants sent to the program, 79 messages (12.7%) were related to NRT. When analyzing the content of these messages, three themes were identified: enthusiasm, instructions, and side effects (Table 3).

**Table 3:** Selected interactions between participants and the tobacco treatment specialist related to NRT management

*Themes*	*Interactions*
**Enthusiasm**	**Male, 31 years old:** Hi! I already started with the patches. It feels good that I have not smoked anything today. **Tobacco treatment specialist:** Congratulations, [Name]! Remember you can text me if you have any questions. Keep on going!
	**Male, 39 years old:** Yes, today I am starting the patches! We will do it! **Tobacco treatment specialist:** Awesome, [Name]! Please text me if you have any questions.
**Instructions**	**Male, 26 years old:** What is the best time to start using the patch? **Tobacco treatment specialist:** [Name], we recommend to wear the patch as soon as you wake up. Please text me if you have any questions.
	**Male, 34 years old:** I have a question, my arm started to sweat and my patch fell off. I tried to place it back on my arm but it would not stay put. Should I apply another one? **Tobacco treatment specialist:** [Name], you can use medical tape to keep the patch in place. Please text me if you have any other questions.
	**Female, 37 years old:** Hello! I was running out of my house today and forgot to put the patch on, will something happen? Should I wear it on my way home or wait until tomorrow? Thank you. **Tobacco treatment specialist:** [Name], we recommend you wear the patch all day long. Is there a way you can get a patch at work? **Female, 37 years old:** No, I live too far from work and I have no one to bring me the patch. I do have some anxiety but I will try my best to not give in since I have not smoked all week. **Tobacco treatment specialist:** Keep strong, [Name]! Remember you can text the keyword Crave for an immediate tip, and make sure to put a patch on as soon as you get home.
	**Male, 36 years old:** I don’t know if this is a psychological thing, but I feel different with the level 2 patches (14 mg patches). I feel like the patches are not working for me. **Tobacco treatment specialist:** [Name], it is normal to feel different with a lower dose of nicotine. We are lowering the dose of nicotine so that your body gets used to less and less nicotine until you don’t need it anymore.
	**Male, 27 years old:** When using the patches, will I need to maintain any specific diet or restrict from consuming any alcohol? **Tobacco treatment specialist:** It is best to stay away from alcohol during the first few weeks after quitting smoking. It will allow you to reduce the temptations and keep your head clear to focus on your quit plan.
**Side effects**	**Female, 30 years old:** Hi, I have a lot of itchiness where I put the patch on. Is that normal? **Tobacco treatment specialist:** [Name], try using the patch in different areas. Make sure the skin area is clean, dry, and hairless. If the itching continues, you can apply an over-the-counter antihistamine cream.
	**Female, 37 years old:** The patches give me insomnia. Hopefully I can rest well tonight. **Tobacco treatment specialist:** [Name], you can take the patch off before sleeping. Remember to put on a new one in the morning.

## DISCUSSION

This study shows preliminary evidence that Mexican smokers, who are eligible to use NRT in accordance with practice guidelines for treating smokers in Mexico, are interested in using NRT for smoking cessation and are willing to engage with a smoking cessation mHealth intervention that aims to increase usage and adherence of NRT. The smoking cessation rate seen at week 12 (end of treatment; 30%, 9/30) appears promising and is in line with end-of-treatment cessation rates seen in trials of NRT that used substantial in-person counseling^25^. In a *b*^n^ randomized factorial trial (*b=2; n=5*) to assess the effects of five intervention components on smokers’ adherence to combined nicotine patch and nicotine gum during a quit attempt, Schlam et al.^22^ found ‘fairly low cessation medication adherence among primary care smokers during the first 6 weeks of a quit attempt’ as only 40% of participants reported using a patch every day. In this pilot study, we found that 40% of participants used the patch <75% of the days at 12 weeks. Future studies should explore the determinants of non-adherence (e.g. educational level, CPD, genetic factors etc.) Moreover, future studies should explore how to gather more proximal and longitudinal real-time data on adherence rather than rely on self-report.

The World Health Organization Framework Convention on Tobacco Control states in Article 14 that ‘each Party [country] shall develop and disseminate appropriate, comprehensive and integrated guidelines based on scientific evidence and best practices, taking into account national circumstances and priorities, and shall take effective measures to promote cessation of tobacco use and adequate treatment for tobacco dependence’^26^. This study demonstrated the feasibility of providing NRT through postal mail in Mexico. The provision of free NRT by postal mail, as done in this study, is ongoing in several countries, including the US and Canada^27-30^. This strategy, when combined with behavioral telephone support, has demonstrated promising results in increasing cessation rates among large populations of smokers^27-30^. As Mexico has less economic resources than the US or Canada, the behavioral over-the-phone support could be delivered via less costly text messages. In this pilot study, participants were able to interact with a tobacco treatment specialist, which represents an additional cost. Future studies should assess the effectiveness and cost-effectiveness of having trained personnel responding to participants’ text messages. However, this study gave us preliminary results on participants’ interactions regarding pharmacotherapy management. These messages can guide the creation of algorithms to recognize and understand natural language to draw expected, automatic responses. For example, the text messaging platform could build a branching logic to recognize the word ‘*itchiness*’ and send as an automated response ‘[Name], *try using the patch in different areas. Make sure the skin area is clean, dry, and hairless. If the itching continues, you can apply an over-the-counter antihistamine cream’.*

Contrary to clinical guidelines^31^ in the United States, NRT is not recommended in Mexico for those who smoke <6 CPD, which represents about 75% of smokers in Mexico^4,12,32^. While reducing tobacco usage is a critical public health priority, only limited research attention has been given to non-daily and light smokers. Non-daily and light smokers have been largely excluded from decades of smoking cessation pharmacotherapy research. Only four pharmacotherapy trials have focused on light smokers and none has included Latinos^33-37^. Consequently, non-daily and light smokers have little evidence to guide them in making choices about effective treatment. As Mexican smokers are more likely to be non-daily and light smokers, Mexico represents a perfect setting for this type of research.

### Limitations

This study had a number of limitations. This was a pilot study and did not have a control group. Follow-up was limited to a single assessment at week 12, when the program ended. Due to the small sample size, the results are not generalizable to all Mexican smokers. Furthermore, the sample was more highly educated than the general population of smokers in Mexico. Future research is warranted to determine whether the effectiveness of this intervention is generalizable to those who are from lower socioeconomic groups. Despite these limitations, the study suggests that managing NRT usage and adherence via text messages is feasible and acceptable and is promising for further testing.

## CONCLUSIONS

Although the role of participant engagement (messages sent to the program by participants) in smoking cessation text messaging programs has received some attention in the tobacco treatment literature^38-40^, more research is needed to evaluate this mechanism. Wang et al.^41^ found that engagement in a chat-based cessation support (interacting with a counselor) through an instant messaging app (WhatsApp) strongly predicted abstinence with or without use of external smoking cessation services. Future studies should assess participant’s engagement with the text messaging program, especially messages related to pharmacotherapy, as potential mediators of pharmacotherapy usage and adherence, and smoking abstinence. The *Vive sin Tabaco… Decídete!* smoking cessation mHealth intervention appears to offer a promising strategy to increase usage and adherence of NRT in Mexico. Additional testing in a formal randomized clinical trial appears warranted.
